# Supplementary material for: Phylogenomics illuminates the backbone of the Myriapoda Tree of Life and reconciles morphological and molecular phylogenies
Source: Sci Rep. 2018 Jan 8;8:83. doi: 10.1038/s41598-017-18562-w (PMC5758774; doi:10.1038/s41598-017-18562-w)
Supplement: Supplementary file 4 — Supplementary Material S4 [file 41598_2017_18562_MOESM4_ESM.doc]

**SUPPL. MAT. S4_MORPHOLOGICAL MATRIX**

#NEXUS

[ File saved by NDE version 0.5.0, Tue Dec 05 10:29:38 2017 ]

BEGIN TAXA;

DIMENSIONS NTAX=29;

TAXLABELS

'Limulus polyphemus'

'Liphistius malayanus'

'Centruroides vittatus'

'Proscorpius osborni'

'Daphnia pulex'

'Rehbachiella kinnekullensis'

'Drosophila melanogaster'

'Folsomia candida'

'Rhyniella praecursor'

'Hanseniella sp.'

'Scutigerella sp.'

'Symphylella sp.'

'Pauropus huxleyi'

'Eudigraphis taiwanensis'

'Glomeris marginata'

'Cyliosoma sp.'

'Casiogrammus ichthyeros'

'Cowiedesmus eroticopodus'

'Brachycybe lecontii '

'Gaspestria genselorum'

'Narceus americanus'

'Crussolum sp.'

'Scutigera coleoptrata'

'Eupolybothrus cavernicolus'

'Devonobius delta'

'Craterostigmus crabilli'

'Mazoscolopendra richardsoni'

'Cryptops hortensis'

'Strigamia maritima'

;

ENDBLOCK;

BEGIN CHARACTERS;

DIMENSIONS NCHAR=187;

FORMAT DATATYPE=STANDARD MISSING=? GAP=- SYMBOLS="01234567";

CHARLABELS

[1] 'Egg tooth on embryonic cuticle of second maxilla'

[2] 'Pattern of segment addition in ontogeny'

[3] 'Number of fully-formed trunk leg pairs in first post-embryonic/post-pupoid stadium'

[4] 'Brood care'

[5] 'Peripatoid and foetoid stadia guarded by mother'

[6] 'Pupoid stage'

[7] 'Nauplius larva'

[8] Holometaboly

[9] 'Structure of appendage on tritocerebral segment'

[10] 'Variability in number of antennal articles'

[11] 'Number of antennal articles'

[12] 'Proportions of antennal articles'

[13] 'Distal part of antenna biramous, with three flagella'

[14] 'Antenna with apical cones'

[15] 'Schaftorgan on antennal scape'

[16] 'Globulus on distal part of ventral antennal branch'

[17] 'Two lateral areas bearing sensilla basiconica on terminal antennal article'

[18] 'Number of cheliceral segments'

[19] 'Plagula ventralis in chelicera'

[20] 'Scorpionid chelate pedipalps'

[21] 'Flattened head capsule'

[22] 'Transverse cephalic suture'

[23] 'Anterior tentorial apodemes'

[24] 'Structure of anterior tentorium'

[25] 'Posterior tentorial apodemes'

[26] 'Fenestrated plate composed of fused transverse tendons of mandibular, mx1, and mx2 segments'

[27] 'Structure of lateral eye'

[28] 'Four ocelli in rhomboid cluster'

[29] 'Number of cellular components in eye'

[30] 'Circumretinular sheath cells'

[31] 'Interocellar sheath cells'

[32] 'Inverted median eyes'

[33] 'Tömösváry organs'

[34] 'Trichobothria innervated by several sensory cells'

[35] 'Cuticle calcification'

[36] 'Entognathy (overgrowth and mandibles and maxillae by cranial folds)'

[37] 'Dentition of labral midpiece / intermediate part'

[38] 'Single transverse seta projecting medially from labral side piece / lateral part'

[39] 'Labral sidepiece / lateral part incised medially'

[40] 'A-shaped epipharyngeal support (labral trapezoid)'

[41] 'Arching of border between labral and clypeal part of epipharynx'

[42] 'Bilobate border between labral and clypeal parts of epipharynx'

[43] 'Single row of bottle-shaped glandular shafts at border between labral and clypeal parts of epipharynx'

[44] 'Single row of bullet-shaped sensilla at proximal margin of field of branching spines at border between labral and clypeal parts of epipharynx '

[45] 'Stomotheca (formed by coxapophyses of palp and leg 1) '

[46] 'Tritosternum / sternapophysis adjacent to palpal coxa '

[47] 'Structure of appendage on post-tritocerebral segment'

[48] 'Four sclerites of mandible intersect at cruciform suture'

[49] 'Mandible composed of two sclerites (lamina condylifera only sclerite differentiated from flank of mandible)'

[50] 'Wide membranous band between sclerites of mandible, Haarpolster on a discrete sclerite'

[51] 'Arrangement of pectinate lamellae on mandible'

[52] 'Dentate lamellae on mandible'

[53] 'Number of groups of teeth in dentate lamella of mandible'

[54] 'Internal and external teeth on mandible'

[55] 'Haarpolster a large lobe with dense, uniform setation'

[56] 'First maxilla'

[57] 'First maxilla coalesced with sternal intermaxillary plate'

[58] 'Gnathochilarial lamellae lingulaes'

[59] 'Median suture on first maxillary coxosternite'

[60] 'Number of articles in telopodite of first maxilla'

[61] '"Curled appendages" along inner margin of telopodite of first maxilla'

[62] 'Brush-like setae along inner margin of telopodite of first maxilla'

[63] 'Plumose setae on coxal process of first maxilla'

[64] 'Maxillary organ'

[65] 'Maxillary nephridia'

[66] 'Limbless postmaxillary segment'

[67] 'Coxae of second maxilla'

[68] 'Metameric pores on second maxillary coxosternum'

[69] 'Form of second maxillary telopodite'

[70] 'Trochanter on second maxilla'

[71] 'Pair of spine bristles at distal end of tibia of second maxilla'

[72] 'Plumose setae on inner surface of tarsus of second maxillary telopodite'

[73] 'Comb-like fringe of setae on distal article of telopodite of second maxilla'

[74] 'Termination of telopodite of second maxilla'

[75] 'Structure of claw of telopodite of second maxilla'

[76] 'Postmaxillary sclerites'

[77] 'Forcipule with fang and venom gland'

[78] 'Pleurite of forcipular segment arching over coxosternite'

[79] 'Forcipular tooth plate'

[80] 'Porodont on forcipular coxosternite'

[81] 'Forcipular coxosternite sclerotised in midline'

[82] 'Forcipular coxosternite deeply embedded into cuticle above second trunk segment'

[83] 'Coxalplatten (forcipular coxosternal apodemes) '

[84] 'Tarsungulum on forcipular/first trunk segment'

[85] 'Spine comb on tarsus of forcipule/first trunk leg'

[86] 'Basal node on forcipular tarsus/tarsungulum'

[87] 'Hinge between articles of forcipular telopodite'

[88] 'Spine bristle on inner edge of forcipular trochanteroprefemur opposed to four spine bristles on each coxal margin'

[89] 'Tergite of forcipular segment '

[90] 'Limb VII as chilaria'

[91] 'Number of post-forcipular leg-bearing segments (in Chilopoda)'

[92] 'Intraspecific variability in number of leg pairs'

[93] 'Hexapod thoracic-abdominal tagmosis'

[94] 'Body segments fused into diplosegments'

[95] 'Fusion of tergites, pleurites and sternites'

[96] '"Special heterotergy" (alternating long and short tergites, with reversal of lengths between seventh and eighth walking leg-bearing segments)'

[97] 'Overlap between tergite 1 and head shield'

[98] 'Second tergite much larger than the following ones'

[99] 'Meso- and metathorax in mature stages bearing wings'

[100] 'Single large tergal plate over trunk segments 7-9'

[101] 'Long tergites divided into two'

[102] 'Intercalary sclerites'

[103] 'Paramedian sutures or grooves on tergum (Längsnähten)'

[104] 'Crescentic sulci on most trunk tergites'

[105] 'Tergite margination'

[106] 'Tergite projections'

[107] 'Large unpaired tergal spines associated with spine bristles, aligned longitudinally on midline'

[108] 'Tergal spicula (hairs)'

[109] 'Paired sternal paramedian pores with valves'

[110] 'Sternal pore areas/sternal glands'

[111] Endosternite

[112] 'Coxal vesicles'

[113] 'Leg pentagonal in cross-section, with marginal spines on the angles'

[114] 'Proliferation of telopodal glands on posterior legs'

[115] 'Socketed spurs D/V, a/m/p on distal extremities of podomeres'

[116] 'Bipartite division of tarsi of anterior series of trunk legs'

[117] 'Tarsi divided into many joints'

[118] 'Tarsal spurs'

[119] 'Tarsus 1 with pair of terminal spurs'

[120] 'Tarsal papillae and resilient sole hairs'

[121] 'Slit sensilla'

[122] Claspers

[123] 'Coxal pouches on legs'

[124] 'Ultimate leg-bearing segment a complete cylinder'

[125] 'Coxopleurites on ultimate legs'

[126] 'Ultimate leg thickened in males'

[127] 'Ultimate leg trochanter'

[128] 'Trochanter of ultimate and penultimate legs with ventral spine'

[129] Furcula

[130] Aculeus

[131] 'Antenna and leg regeneration'

[132] 'Relationship of sternal and lateral longitudinal muscles'

[133] 'Position of spiracle(s)'

[134] Anisostigmophory

[135] 'Shape of stigmatic plates'

[136] 'First and second stigmatic plate'

[137] 'Longitudinal and transverse connections between segmental tracheal branches'

[138] Chiasmata

[139] 'Arrangement of tracheal pouches in comparison to body axis'

[140] 'Apodemes of mid-body tracheal pouches'

[141] 'Branching of mid-body tracheal pouch apodemes'

[142] 'Foregut with differentiated gizzard with plicate walls'

[143] 'Posterior part of foregut organised as a sieve with stiff, anteriorly directed projections'

[144] 'Midgut developed within the yolk'

[145] 'Intestine shape'

[146] 'Malpighian tubules formed as endodermal extensions of midgut'

[147] 'Malpighian tubules formed as ectodermal extensions of hindgut'

[148] 'Lateral defense glands'

[149] 'Shape of defence glands'

[150] 'Defence secretions with benzoquinines'

[151] 'Testes differentiated into macrotestis with ampulla and microtestis'

[152] 'Lateral testicular vesicles linked by a central, posteriorly extended deferens duct'

[153] 'Testicular vesicles spindle shaped'

[154] 'Female gonopod on first genital segment'

[155] 'Female gonopod used to manipulate single eggs'

[156] 'Female gonopod segmentation'

[157] 'Female gonopod with basal article bearing spurs (macrosetae) and terminal article with a broad claw'

[158] 'Claw of female gonopod fused with the apical article'

[159] 'Position of male gonopore'

[160] 'Segmentation of male gonopod on first genital segment'

[161] 'Male gonopod on second genital segment'

[162] 'Form of male gonopod on second genital segment '

[163] 'Bivalved anogenital capsule'

[164] 'Anal organs'

[165] 'Coxal organs'

[166] 'Serial distribution of coxal organs'

[167] 'Arrangement of coxal pores'

[168] 'Spermatophore web produced by Spinngriffel on first genital segment of male'

[169] 'Bean-shaped spermatophore with tough, multi-layered wall'

[170] 'Sperm dimorphism'

[171] 'Sperm flagellum'

[172] 'Coiling of sperm flagellum around nucleus'

[173] 'Spiral ridge on nucleus of sperm'

[174] 'Sperm nucleus with machette of microtubules'

[175] Pseudoperforatorium

[176] 'Spermathecae formed by paired pockets in mouth cavity'

[177] 'Modified leg(s) in male on seventh and/or eight trunk segment(s)'

[178] 'Leg 8 in adult males'

[179] 'Leg 9 in adult males'

[180] 'Leg 10 in adult males'

[181] 'Posterior male leg pair as telopods with inner horns'

[182] 'Pre-anal segment with trichobothria bearing a long sensory seta'

[183] 'Appendage on opisthosomal segment I '

[184] 'Lamellate respiratory organs derived from posterior wall of trunk limb buds'

[185] 'Type of lamellate opisthosomal respiratory organs'

[186] 'Thoracic food groove (invagination of sternites) and filter-feeding apparatus'

[187] 'Serrate setae arranged in lateral and caudal tufts'

;

STATELABELS

1

absent

present,

2

hemianamorphosis

euanamorphosis

epimorphosis,

3

three

'four (Platydesmida coded as groundpattern)'

'forcipule + four'

'forcipule + 12',

4

absent

'female (male in Brachycybe) bends ventrally around eggs'

'female bends dorsally around eggs',

5

absent

present,

6

absent

present,

7

absent

present,

8

absent

present,

9

antenna

'chelicera ',

10

'variable within the species'

'fixed within the species',

11

'15 or more'

'14'

'eight or fewer',

12

'at least some approximately as long as wide'

'consistently ring-like, much wider than long',

13

absent

present,

14

'apical cones absent'

'apical cones present',

15

absent

present,

16

absent

present,

17

absent

present,

18

three

two,

19

absent

present,

20

absent

present,

21

'head capsule domed'

'flattened, with head bent posterior to the clypeus',

22

'absent '

'present (frontal line or frontal sulcus)'

'present, divided near lateral margin into antenocellar suture',

23

absent

present,

24

'separate, rod-like anterior tentorial arms'

'an unpaired roof',

25

absent

'present ',

26

absent

present,

27

stemmata

'compound / facetted'

'single ocellus'

absent

'simple lens with cup-shaped retina',

28

'absent '

present,

29

'facultative constancy'

'all cellular components highly variable in number',

30

absent

present,

31

absent

present,

32

absent

present,

33

absent

present,

34

absent

present,

35

absent

present,

36

absent

present,

37

'undefined midpiece (median tooth / teeth lacking)'

'single strong tooth'

'three teeth'

'several (>3) small teeth ',

38

absent

present,

39

'not incised '

incised,

40

absent

present,

41

'subtransverse or gently arched distally'

'strongly arched distally',

42

absent

present,

43

absent

present,

44

absent

present,

45

absent

present,

46

'absent (coded for post-oral somite II)'

present,

47

'pedipalp or locomotory leg'

mandible,

48

absent

present,

49

absent

present,

50

absent

present,

51

'multiple imbricated lamellae'

'lamellae arranged in a single file',

52

present

absent,

53

three

'four/five',

54

absent

present,

55

absent

present,

56

'absent (segmentally homologous appendage a locomotory limb)'

present,

57

absent

'present, with unfused stipital and intermaxillary components '

'mental elements of gnathochilarium consolidated',

58

separate

'connected ("fused")',

59

'coxae medially coalesced, separated by median suture'

'coxae fused, without median suture',

60

two

one,

61

absent

present,

62

absent

'with short, curved barbs '

'plumose, branching as slender hairs',

63

absent

present,

64

absent

present,

65

paired

fused

absent,

66

'limbs present'

'limbs absent',

67

'coxae separate'

'coxae fused',

68

'minute opening of second maxillary gland medial to mx2 coxosternite'

'enlarged opening of mx2 gland ("metameric pore") incorporated in medial part of mx2 coxosternite'

'metameric pore on lateral part of mx2 coxosternite',

69

'slender, leg-like, with elongate prefemur/femur'

'short, stout',

70

present

absent,

71

absent

present,

72

'absent (simple setae)'

'plumose setae present',

73

absent

'bifurcating of multifurcating spines'

'densely-aligned simple bristles',

74

'simple (no claw or seta)'

claw

'seta or setigerous tubercle',

75

'unipartite, conical claw'

'thick, elongate digits with interspersed thin digits'

'hook-like claw',

76

absent

present,

77

absent

'present (fossils coded based on fang)',

78

'absent (small pleurite)'

'pleurite arching over coxosternum, discontinuous medially ("Spange" of Attems, 1926)'

'pleurite arching over coxosternum, continuous ventromedially',

79

absent

'anteriorly projecting serrate endite'

'transverse sclerotised band on anterior margin',

80

'absent '

'translucent, seta-like porodont',

81

'coxae separated medially, with sternite present in adult'

'coxosternal plates meeting medially, with flexible hinge'

'coxosternal plates meeting medially, hinge sclerotised and non-functional',

82

'not embedded'

'deeply embedded',

83

absent

present,

84

'separate tarsus and pretarsus'

'tarsus and pretarsus fused',

85

absent

present,

86

'absent (coded for first trunk leg in Progoneata and Hexapoda)'

present,

87

'between prefemur/trochanteroprefemur and femur'

'between trochanteroprefemur and tibia'

'between trochanteroprefemur and tarsungulum',

88

absent

present,

89

'separate tergite'

'separate tergite lacking, fused to next posterior segment',

90

absent

present,

91

'15'

'21'

'47-51',

92

'constant number of leg pairs'

'variable number of leg pairs',

93

absent

present,

94

absent

present,

95

'absent (free pleurites and sternites)'

'pleurotergites (pleurae fused to tergites, with free sternites)'

'complete body rings (tergites, pleurites and sternites fused)',

96

absent

present,

97

'head shield overlaps tergite 1'

'tergite 1 overlaps head shield',

98

absent

present,

99

absent

present,

100

'separate tergites'

'single tergite',

101

absent

present,

102

'absent or weakly sclerotised'

'small intercalary tergites (pretergites) and sternites'

'strongly developed intercalary tergites and sternites',

103

absent

present,

104

absent

present,

105

'absent or on last tergite only'

'on most or all tergites',

106

'absent on all tergites'

'present on at least TT11 and 13',

107

absent

present,

108

absent

present,

109

absent

present,

110

'absent in at least female'

'present in both sexes',

111

absent

present,

112

absent

'present at limb base on numerous trunk segments'

'on distal part of first abdominal segment as ventral tube',

113

absent

present,

114

absent

present,

115

absent

present,

116

absent

present,

117

'tarsi undivided or bisegmented'

'tarsus flagelliform, with many joints',

118

absent

present,

119

absent

present,

120

absent

present,

121

absent

present,

122

absent

'present (on anterior thoracopod)',

123

absent

present,

124

absent

present,

125

'coxa and pleurites fused as short coxopleurite'

'elongate coxopleurite',

126

'inconspicuous sexual dimorphism with respect to proportions of ultimate leg'

present,

127

present

'minute or absent',

128

absent

present,

129

absent

present,

130

absent

present,

131

present

absent,

132

'united sternal and lateral longitudinal muscles'

'separate sternal and lateral longitudinal muscles, with separate segmental tendons',

133

absent

pleural

'dorsal opening on tergum '

'sternal, at base of legs, with tracheal pouch serving as apodeme'

'single spiracle, opening on head'

'single spiracle on opisthomsomal segment 2',

134

'absent (spiracles present on all trunk segments from second pedigerous segment)'

'present (spiracles associated with long tergites only)',

135

'flat, triangular'

'divided in midline'

'sterna nodifera sensu Blanke and Wesener (2014, character 20)'

'sliding sternites sensu Blanke and Wesener (2014, character 20)',

136

'with tracheae'

'without tracheae, used for muscle attachment',

137

absent

present,

138

absent

present,

139

'diagonal orientation'

'longitudinal orientation',

140

absent

'present, serving as muscle attachment',

141

uniramous

biramous,

142

absent

present,

143

absent

present,

144

'midgut cells enclose entire yolk'

'lumen of embryonic midgut lacking yolk globules',

145

'straight tube'

'N-shaped, bent twice',

146

absent

present,

147

absent

present,

148

absent

present,

149

'elongate subtubular'

subspherical,

150

absent

'present (coded as grounpattern for Juliformia)',

151

present

absent,

152

absent

present,

153

absent

present,

154

absent

present,

155

absent

present,

156

'three articles and claw, with basal articles of gonopod pair separated'

'two articles, the proximal article of each gonopod pair partly joined (syntelopodite), the distal article a spine'

'single segment or with rudimentary second segment',

157

absent

present,

158

'claw separate'

'claw fused',

159

opisthogoneate

'behind coxa of second pair of trunk legs'

'through coxa of second pair of trunk legs'

'on fourth trunk segment',

160

'two segments'

'single segment, rudimentary'

'unsegmented style',

161

present

absent,

162

'blunt cones'

'slender styles ',

163

absent

present,

164

absent

'present through ontogeny'

'present only in juveniles',

165

absent

present,

166

'on last four pairs of legs'

'on last pair of legs only',

167

'few pores in linear row'

'numerous small pores scattered over coxopleure or large pore field'

'opening in depressions between meshwork of ridges',

168

absent

present,

169

absent

present,

170

absent

'microsperm and macrosperm present',

171

present

absent,

172

'absent (filiform)'

present,

173

absent

present,

174

absent

present,

175

absent

present,

176

absent

present,

177

absent

present,

178

'walking leg (unmodified)'

'accessory gonopod'

'functional gonopod',

179

'walking leg (unmodified)'

'accessory gonopod'

'functional gonopod',

180

'walking leg (unmodified)'

'functional gonopod',

181

absent

present,

182

absent

present,

183

'present (coded for eighth limb-bearing metamere)'

absent,

184

absent

present,

185

'book gills'

'book lungs',

186

absent

present,

187

absent

present,

;

MATRIX

'Limulus polyphemus' ?2-000001- -------000 0?0-0?10?? ?00000???? ????000--- -----0---- ---0?????? ??????0--- ---??????1 ?00000??00 -????????? ?00?0????0 00?0????00 ??0------- -0??0000-- ???0----?- --000--0-0 0000000??? 0001000

'Liphistius malayanus' ?2-0?0001- -------110 ??0-0?4??? ?10100???? ????010--- -----0---- ---0?????? ??????0--- ---??????0 ?00000??00 -????????? ?00?0????0 10?0????00 ??0------- -0??0100-- ???0----?- --000--0?0 0111000??? 0011100

'Centruroides vittatus' ?2-0?0001- -------001 ??0-0?4??? ?10100???? ????100--- -----0---- ---0?????? ??????0--- ---??????0 ?00000??00 -????????? ?00?0????0 10?0????01 ??0------- -???0100-- ???0----?- --000--0?0 0000000??? 0011100

'Proscorpius osborni' ????????1- -------0?1 ??????4??? ????00???? ????0?0--- -----0---- ---??????? ??????0--- ---??????0 ??00?0??00 -????????? ?00?0????? ???0????01 ?????????? ?????????? ?????????? ??0??????? ??????0??? 0?1??00

'Daphnia pulex' ?0??00100? ??00000--- ??0-0?10?? ?00000???? ????00100? -???-10-?? ???0?0???? ??????0--- ---??????0 ??0000??00 -????????? ?00?0????0 01?0????00 ??0------- -0???000-- ???0----?- --000--0-0 1-00000??? 0000-10

'Rehbachiella kinnekullensis' ??????100? ??00000--- ??????10?? ????00???? ????0?100? -???-10-?? ?????0???? ??????0--- ---??????0 ??0000??00 -????????? ?00?0????0 ?0?0????00 ??0------- -????????? ???0----?? ??0?0--??? ??????0??? 0000-10

'Drosophila melanogaster' 02-0000101 ?000000--- 0?111010?? ?00000???? ????00---- -----10-?? ??-020???? ??????0--- ---0?0???0 ?01000?010 -????????? ?00?0?00?0 00?0????00 ??1---1?-- -??00010-- ???0----0- --000--0-0 ?000000??? 0000-00

'Folsomia candida' 02-0000001 2000000--- 0?101?3--- -01001???? ????001000 -0?0-10-?? ??-000???? ??????0--- ---0?????0 ?01000?000 -????????? ?20?0?00?0 00?0????10 ??0------- -???0000-- ???0----0- --000--0?? ?????????? ??00-00

'Rhyniella praecursor' ????????0? 200????--- 0?????10?? ????01???? ????0?1??0 ?0??-????? ?????0???? ??????0--- ---0?????0 ??1000??00 -????????? ?20?0?00?0 ???0????1? ?????????? ?????????? ?????????? ?????????? ?????????? ??00-?0

'Hanseniella sp.' 00??000000 0000000--- 00100?3--- --10000000 ????0010?0 -0?0-10-?- ---000---- -----?0--- ---0000000 ?00000?000 -?00000000 0100000000 00?0-0?000 ??4------- -??10010-- ???0----3- --000--00? ?????10000 0100-00

'Scutigerella sp.' ?0??0?0000 0000000--- 00100?3--- --10000000 ????0010?0 -0?0-10-?- ---000---- -----?0--- ---0000000 ?00000?000 -?00000000 0100000000 00?0-0?000 ??4------- -???0010-- ???0----3- --000--001 0000010000 0100-00

'Symphylella sp.' ?0????0000 0000000--- 00100?3--- --10000000 ????0010?0 -0?0-10-?- ---0?0---- -----?0--- ---0000000 ?00000?000 -?00000000 0100000000 00?0-0?000 ??4------- -???00?0-- ???0----3- --000--??? ?????10000 0100-00

'Pauropus huxleyi' -000010001 2010010--- 0010003--- --1000?000 ????0010?0 -??0-110?- ---001---- -----?0--- ---0000000 ?00000?000 -?00000000 0000010000 00?0-0?000 ??0------- -??10010-- ???0----1- --000--00? 000000000- 0000-00

'Eudigraphis taiwanensis' -000010001 2001000--- 00100?1000 0-10000000 ????001000 00?0-110?- ---0?1---- ------0--- ---0000000 ?00100?000 -000?00000 0000010000 0010-0?000 ?03-000000 -0??0010-- ???0----1- --000--0?? ?????00000 0000-01

'Glomeris marginata' -000010001 2001000--- 0010000000 0-10101000 ????001000 00?1-121?- ---001---- -----?0--- ---0000000 ?00100?100 -000?00000 0000000000 0000-??000 ?03-101001 10?11010-0 ???0----1- --000--000 1-00000000 1000-00

'Cyliosoma sp.' -0?00?0001 2001000--- 00100000?? ?-10101000 ????001000 00?1-121?- ---0?1---- ------0--- ---0000000 ?00100?100 -000?00000 0000000000 0000-??000 ??3-101001 1???1010-- ???0----1- --000--0?0 1-00000000 1000-00

'Casiogrammus ichthyeros' ?????????? ?????????? ?????????? ????1????? ?????????? ?????????? ?????????? ?????????? ?????????? ??0100??00 -000?000?? ?????????? ?????????? ?????????? ?????????? ?????????? ?????????? ?????????? ???????

'Cowiedesmus eroticopodus' -????????? ???????--? 0????????? ????10???? ?????????? ?????????? ?????????? ??????0--- ---?????00 ??0110?000 -0???00?1? ?00?0?000? ?????????? ?????????? ?????????? ?????????? ?????????? ??????1000 ??0???0

'Brachycybe lecontii ' -1110?0001 2001000--- 00100?3--- --001000?0 ????001?00 00?1-120?- ??-0?1---- -----?0--- ---0000000 ??0110?000 -000?00000 0000000000 0010-??000 ??3-210001 0???00110? ???0----1- --000--0?? ?????01011 0000-00

'Gaspestria genselorum' -????????? ?????????? 0????????? ????10???? ?????????? ???1?????? ??????---- -----????? ????????00 ??0120?000 -000?000?? ?00?0?00?? ????????00 ?????????? ???????1?? ?????????? ?-???????? ?????????? 0?00-00

'Narceus americanus' -100010001 2001000--- 00100000?? ?-001020?0 ????001000 00?1-120?- ---001---- ------0--- ---0000000 ?10120?000 -000?00000 0000000000 0000-??000 ?03-310011 00??001111 ???0----1- --000--0?0 1-00101120 0000-00

'Crussolum sp.' ?????????? ??0??????? ?????????? ????00???? ?????????? ?????????? ?????????? ??????1?0? 00????00?? ??0?????0? ?????????? ?01?001000 ?????????? ?????????? ?????????? ?????????? ?????????? ?????????? ??????0

'Scutigera coleoptrata' 1020000000 0100100--- 0110011-00 0-10001001 0000001001 0000010-00 0101100000 1000-01000 0000100100 0000010001 0000001100 0010011011 0000000000 0021--00-- -0000010-- 00-1110-02 01000--001 0010000000 0000-00

'Eupolybothrus cavernicolus' ?0?00?0000 0000000--- 1210000011 1-10001110 0010001010 0010010-00 0210?01001 0101111001 1011000000 0000010000 0000110000 0001110000 0000000000 0011--00-- -?0?0010-- ??-1101102 1-021011?? ?????00000 0000-00

'Devonobius delta' ????????0? ?0000?0--- 11????3--- -?0?00???? ????0?1??? ?0????0??? ?????0100? ??????1110 2111010000 ??0001?000 0?00000000 100?01000? ?????0??0? ??1?--???? ?????????? ?????????? ????1????? ??????0??? ??00-00

'Craterostigmus crabilli' ?0310?0001 0000000--- 1210002-11 0-10003000 ?000001010 ?000110-01 0000201001 0011111110 2111000000 0000010000 1100000000 0000000100 0001100100 ?111--00-- -???0010-- ???0----01 1-111121?? ?????00000 0000-00

'Mazoscolopendra richardsoni' ????????0? 000????--- 1????????? ????00???? ????0?1??? ?????1???? ?????????? ??????1??? ???10?2010 100001?000 0????00??? ?00?0?0??? ?0?01???00 ?????????? ?????????? ?????????? ??0?111??? ??????0000 0?00-00

'Cryptops hortensis' 12-1100001 00000-0--- 1010003--- --00001000 0001001100 0010010-00 1000201-01 0021201120 2111001010 1000011000 0211000000 1000000000 0000101000 0111--10-- -11?0010-- ???0----0- 1-0011111? 0010000000 0000-00

'Strigamia maritima' 12-2100001 10000-1--- 1010003--- --00003000 1100001000 11---10-10 0000201111 0001001100 2111012000 2100000000 -200000001 0000000000 0000110000 1110--11-- -00?0010-- 1111020-00 1-011111?0 0010000000 0000-00

;

ENDBLOCK;

BEGIN ASSUMPTIONS;

OPTIONS DEFTYPE=UNORD POLYTCOUNT=MINSTEPS;

TYPESET * default = ORD: 57 68 81 95 102;

ENDBLOCK;

BEGIN NOTES;

[Taxon comments]

[Character comments]

[Character state comments]

[Attribute comments]

TEXT TAXON=14 CHARACTER=97 TEXT='check under microscope';

TEXT TAXON=28 CHARACTER=4 TEXT='Coding based on C. anceps (Brunhuberg, 1970) and C. multispinus (Lawrence, 1947)';

TEXT TAXON=29 CHARACTER=1 TEXT='Lewis 1961';

[Taxon pictures]

[Character pictures]

[Character state pictures]

[Attribute pictures]

ENDBLOCK;
